# Implicating the Role of GILZ in Glucocorticoid Modulation of T-Cell Activation

**DOI:** 10.3389/fimmu.2019.01823

**Published:** 2019-08-07

**Authors:** Lorenza Cannarile, Domenico V. Delfino, Sabrina Adorisio, Carlo Riccardi, Emira Ayroldi

**Affiliations:** Section of Pharmacology, Department of Medicine, Medical School, University of Perugia, Perugia, Italy

**Keywords:** glucocorticoids, glucocorticoid-induced leucine zipper (GILZ), T-cell activation, T-cell, immune response, glucocorticoid receptor

## Abstract

Glucocorticoid-induced leucine zipper (GILZ) is a protein with multiple biological roles that is upregulated by glucocorticoids (GCs) in both immune and non-immune cells. Importantly, GCs are immunosuppressive primarily due to their regulation of cell signaling pathways that are crucial for immune system activity. GILZ, which is transcriptionally induced by the glucocorticoid receptor (GR), mediates part of these immunosuppressive, and anti-inflammatory effects, thereby controlling immune cell proliferation, survival, and differentiation. The primary immune cells targeted by the immunosuppressive activity of GCs are T cells. Importantly, the effects of GCs on T cells are partially mediated by GILZ. In fact, GILZ regulates T-cell activation, and differentiation by binding and inhibiting factors essential for T-cell function. For example, GILZ associates with nuclear factor-κB (NF-κB), c-Fos, and c-Jun and inhibits NF-κB-, and AP-1-dependent transcription. GILZ also binds Raf and Ras, inhibits activation of Ras/Raf downstream targets, including mitogen-activated protein kinase 1 (MAPK1). In addition GILZ inhibits forkhead box O3 (FoxO3) without physical interaction. GILZ also promotes the activity of regulatory T cells (Tregs) by activating transforming growth factor-β (TGF-β) signaling. Ultimately, these actions inhibit T-cell activation and modulate the differentiation of T helper (Th)-1, Th-2, Th-17 cells, thereby mediating the immunosuppressive effects of GCs on T cells. In this mini-review, we discuss how GILZ mediates GC activity on T cells, focusing mainly on the therapeutic potential of this protein as a more targeted anti-inflammatory/immunosuppressive GC therapy.

## Introduction

Glucocorticoids (GCs) are the mainstay of current immunosuppressive and anti-inflammatory therapies (1). Decades of study have revealed that their primary mechanism of action involves GC binding to GC receptors (GRs) to modulate gene transcription (2–5). However, the biological effects of GCs are diverse and are likely controlled by several mechanisms. Given this functional diversity, identifying molecules that are transcriptionally induced by GCs, and can mediate specific GC effects presents a significant challenge.

One potential molecule is glucocorticoid-induced leucine zipper (GILZ), a ubiquitously expressed protein that is primarily under GR transcriptional control. GILZ was originally identified in 1997 when searching for genes that mediate GC-induced apoptosis (6). However, since that time, the roles of GILZ have expanded to include most of the anti-inflammatory, and immunosuppressive effects of GCs (7). Indeed, GILZ is now known to regulate cell apoptosis, proliferation, and differentiation by modulating transcription factors, and signaling pathways associated with host immunity, and inflammation (8–12).

GILZ has a high degree of homology with other members of the TSC22D family. The TSC22D family includes leucine zipper proteins that are differentially expressed and involved in the regulation of multiple biological processes (13). TSC22D isoform heterodimers regulate cell cycle entry and exit (14).

One mechanism by which GCs induce immunosuppression is through regulation of the T-cell response (15, 16). In this review, we discuss the literature concerning how GILZ mediates the effects of GCs on T cells. Regardless of the specific role of GILZ, we highlight information about GC-dependent, and GC-independent GILZ functions to expand the current understanding of the GC mechanism of action. Ultimately, such understanding is critical to improving GC clinical use.

## GCs and the T-Cell Response

**Figure 1:**
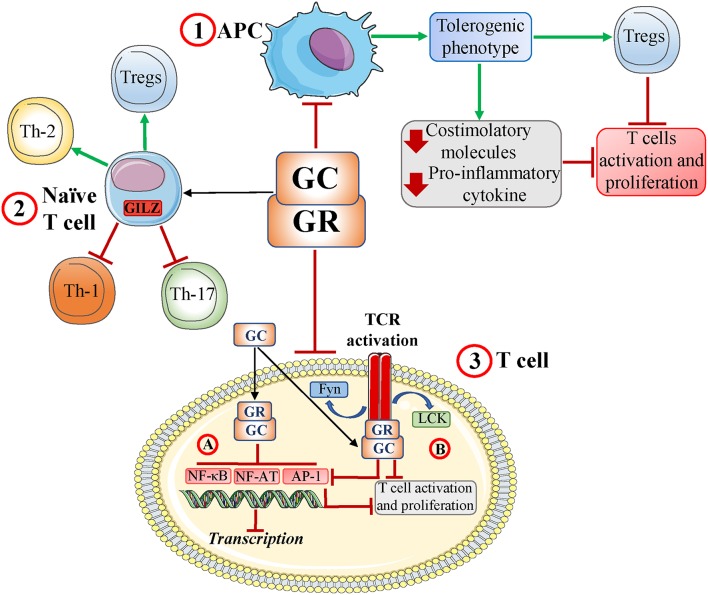
Glucocorticoids and the T-cell response. Glucocorticoid **(**GCs) and glucocorticoid receptor (GR) interactions induce: (1) a tolerogenic antigen-presenting cell (APC) phenotype with decreased production of both proinflammatory chemokines and costimulatory molecules and development of regulatory T cells (Tregs). This subsequently inhibits T-cell activation; (2) a modulation of naïve T-cell differentiation, including inhibition of Th-1 and Th-17 cell development and induction of Th-2 cells and Tregs; and (3) inhibition of T-cell receptor (TCR) signaling by inhibiting (genomic effects) key transcription factors such as NF-AT, AP-1, and NF-κB (3A) and disruption of TCR-associated multiprotein complexes containing GR, LCK, and FYN (rapid, non-genomic effects) with inhibition of NF-AT, AP-1, and NF-κB (3B). Ultimately, these interactions impair TCR signaling and T-cell activation/proliferation. Red T-headed leaders indicate inhibition; green arrow-headed leaders indicate activation.

T-cell activation is an essential part of the adaptive, cell-mediated immune response. GCs modulate T-cell differentiation and activation regulating: (1) antigen-presenting cells (APCs); (2) T helper (Th) cell differentiation; and (3) T-cell receptor (TCR) signaling (Figure 1) (15). The GR acts through genomic and non-genomic mechanisms, regulating adhesion molecules, co-accessory molecules, and cytokines implicated in T-cell activation (17–19).

Acting directly on T cells, GCs function through different mechanisms, most of which involve GR/transcription factor interaction. GCs affect the activity of transcription factors downstream of TCR activation, including nuclear factor-κB (NF-κB), activator protein-1 (AP-1), and nuclear factor of activated T cells (NF-AT) (15). GCs can also act through non-genomic mechanisms to limit kinase activity downstream of TCR activation, ultimately inhibiting the above-mentioned transcription factors and T-cell activation (20) (Figure 1).

Moreover, GCs can modulate T-cell activation indirectly through other cells such as dendritic cells (DCs), which are professional APCs. DCs have dual functionality, as they both orchestrate adaptive immune responses and also actively maintain peripheral specific tolerance against innocuous antigens (21). The balance between the activating and tolerogenic DC phenotypes is crucial to generating an efficient immune response while also preventing autoimmunity. GCs inhibit DC functions, reducing expression of MHC class II, and costimulatory molecules, decreasing proinflammatory cytokines and increasing anti-inflammatory cytokines such as IL-10 (22). Importantly, GCs can also increase the ability of DCs to capture antigens, suggesting that GCs drive DCs toward a tolerogenic phenotype (23). Tolerogenic DCs induce T-cell suppression and anergy and promote the generation of regulatory T cells (Tregs) (24). Therefore, GC modulation of DCs indirectly inhibits T-cell activation (Figure 1).

GCs can also modulate T cells by targeting tissue macrophages, mast cells, and stromal cells. Myeloid cells modulate T-cell function, acting as APCs and/or secreting inflammatory cytokines in response to stimulation of pattern recognition receptors (PRRs) (15). GCs can attenuate signals downstream of PRR activation, including the transcription factors AP-1, NF-κB, and the mitogen-activated protein kinase 1 (MAPK1) pathway (15, 25, 26). Those signaling changes alter the cytokine network, with important consequences for both inflammation, and T-cell responses. In fact, this mechanism may partially account for both GC inhibition of Th-1 and Th-17 differentiation and GC promotion of Th-2 differentiation and Treg production (27, 28) (Figure 1).

What is the role of GILZ in this context?

## GILZ and the T-cell Response

**Figure 2:**
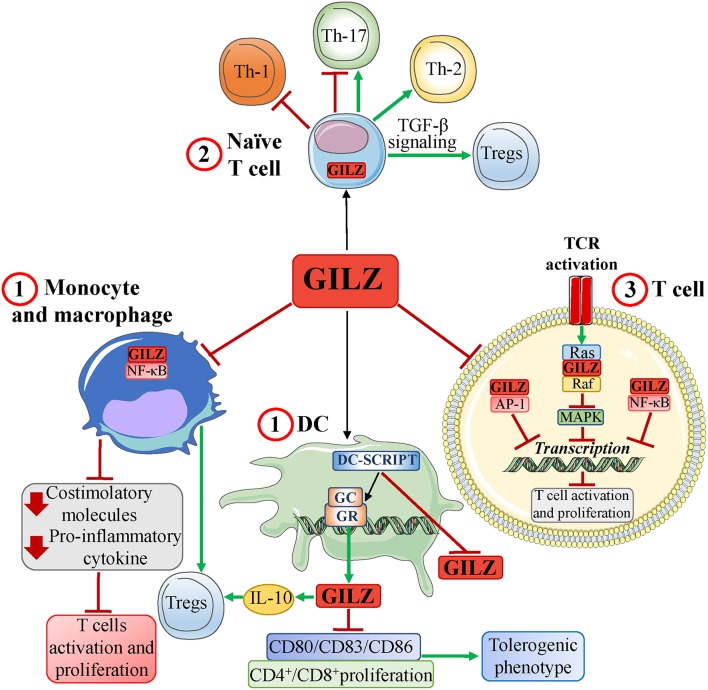
GILZ and the T-cell response. GILZ, expressed basally and/or in response to GCs, induces: (1) a tolerogenic dendritic cell (DC) phenotype and inhibition of human monocyte and mouse macrophage activation via inhibition of NF-κB, thereby limiting production of both proinflammatory chemokines, and costimulatory molecules. In DCs, GILZ expression is regulated by a GR corepressor, DC-specific transcript (DC-SCRIPT), whose recruitment inhibits GILZ expression. GILZ-induced inhibition of APC functions promotes development of Tregs and ultimately inhibits T-cell activation; (2) a modulation of naïve T-cell differentiation (expressing endogenous GILZ) that includes induction of Th-2 cells and Tregs (favoring TGF-β signaling), inhibition of Th-1 cells, and inhibition or development of Th-17 cells; (3) an inhibition of TCR signaling by inhibiting pathways, such as MAPK, and transcription factors, such as AP-1, and NF-κB, through protein-protein interactions. Red T-headed leaders indicate inhibition; green arrow-headed leaders indicate activation.

Similar to the GCs, GILZ inhibits innate, and adaptive immune responses, affecting T-cell function (activation, differentiation, and apoptosis) either directly or through APCs (7, 9, 10) (Figure 2).

GC-induced GILZ expression in T cells is involved in multiple GC effects (9); however, its endogenous expression in the naïve T cell suggests a GC-independent function (29).

GCs can modulate T-cell apoptosis, and GILZ can either induce or protect against apoptosis (6). The first studies on apoptosis were performed using the T-cell hybridoma 3DO, which overexpresses GILZ (6, 30). In this cell line, GILZ inhibits both NF-κB (30), and AP-1 (31), behaves as a GC by inhibiting CD3-mediated apoptosis and TCR-driven IL-2 production through Fas/FasL modulation (30). Furthermore, T cells from GILZ-knockout mice (GILZ-KO) show increased antigen-induced T-cell activation (32). These data indicate mutual antagonism between GILZ expression and T-cell activation, suggesting that T cells must inhibit GILZ expression to become activated (29, 33). Moreover, in T cells, GILZ expression mimics the antiproliferative effects of GCs by interacting with Ras and Raf and inhibiting Ras downstream signals, such as MAPK (33, 34) (Figure 2). Notably, IL-2 deprivation in T cells upregulates GILZ (35), whereas IL-2 treatment (35), and T-cell activation (29, 30, 33) decrease GILZ expression. Moreover, IL-2 withdrawal induces cell death and upregulates GILZ by promoting forkhead box O3 (FoxO3) transcriptional activity in GILZ promoter region. In turn, GILZ prevents FoxO3 transcriptional activity, promoting its nuclear exclusion through a mechanism involving the nuclear export receptor Crm1 (36), and inhibiting its own expression and that of the proapoptotic gene Bim. In this case, GILZ protects T cells from IL-2 withdrawal-induced apoptosis by regulating its own expression (35, 37). The role of GILZ in T-cell apoptosis has been further clarified using GILZ transgenic mouse models (GILZ-TG). Thymocytes from GILZ-TG mice undergo apoptosis through caspase-8 activation and Bcl-xL downregulation (38), regulating the thymic repertoire similar to GCs. However, these cells are rescued by TCR-induced apoptosis, suggesting a GC-like mechanism of mutual exclusion (39). In contrast, GILZ does not induce apoptosis in peripheral mature mouse T lymphocytes (40). The ability of GCs to induce the apoptosis of lymphoid cells supports their inclusion in protocols for the treatment of lymphohematopoietic malignancies. GILZ upregulation may underlie these effects of GCs. For example, in multiple myeloma, for which GCs are used, decreasing GILZ levels by siRNA knockdown inhibited GC-induced apoptosis (41).

Constitutive expression of GILZ in naïve T cells (29) plays a major role in their differentiation (Figure 2). GILZ promotes Treg differentiation by activating transforming growth factor-β (TGF-β) signaling (42) and is partly responsible for GC-mediated effects on Tregs (15). In fact, dexamethasone (DEX) treatment augments the frequency of splenic Tregs in WT, but not GILZ-KO, mice (42).

Moreover, GILZ overexpression in CD4+ lymphocytes from GILZ-TG mice promotes Th-2 and inhibits Th-1 differentiation (43); thus, GILZ behaves like GCs (27, 44). As a consequence, GILZ-TG mice are less susceptible to Th-1-mediated diseases, such as experimental dinitrobenzene sulfonic acid- (DNBS-) colitis (45), and spinal cord injury (46). In these models, GILZ-TG mice exhibit an attenuated immune response, which may be explained by GILZ-mediated inhibition of NF-κB, which is crucial for Th-1 cytokine production, in T cells of the intestinal lamina propria, and in spinal cord lesions, respectively (45, 46). Accordingly, injection of mice with either the transactivator of transcription (TAT)-glutathione-*S*-transferase (GST)-GILZ (TAT-GST–GILZ) fusion protein or high doses of DEX, which upregulates GILZ in mucosal T lymphocytes, rescues mice from Th-1-mediated experimental colitis, again by inhibiting NF-κB (45). GILZ, in this model, is crucial for effects on Tregs cells. In fact, in GILZ-KO mice, the severity of DNBS-colitis is increased compared with WT due to impaired generation of Tregs cells. Transfer of WT Treg cells reverses the augmented vulnerability. DEX ameliorates the symptoms of DNBS-colitis in WT, but not GILZ-KO, through Treg augmentation. Therefore, GC anti-inflammatory activities in this model may be mediated by GILZ expression in T lymphocytes (45), and GILZ-induced Treg generation (42). However, in other murine models of inflammation, GILZ does not appear to be involved in the anti-inflammatory activity of GCs. For example, endogenous GILZ is detectable in the synovia of mice with collagen-induced arthritis (CIA), and in patients with active rheumatoid arthritis, and is upregulated by GC therapeutic doses (47). Moreover, GILZ reduction by RNAi worsens the symptoms of CIA, suggesting a role for GILZ as an endogenous inhibitor (47). However, its deletion does not impair the effects of exogenous GCs in CIA and does not affect the severity of antigen-induced or K/BxN serum–transfer arthritis (32). In fact, no difference in arthritis severity was found between GILZ-KO and WT mice, although antigen-induced T-cell proliferation was higher in GILZ-KO mice. However, injection of adeno-associated virus expressing GILZ (GILZ-rAAV) in CIA mice results in joint GILZ expression and attenuation of joint inflammation without affecting T-cell proliferation (32). These data suggest different roles for GILZ in inflammation, again as a brake for T-cell proliferation, an endogenous natural anti-inflammatory protein, and as a drug.

A pharmacological use of the GILZ protein was also shown in experimental autoimmune encephalomyelitis (EAE), an inflammatory model for human multiple sclerosis. GILZ peptide (GILZ-P) binds to and inhibits NF-κB, suppresses T-cell activation, and shows therapeutic efficacy when administered in EAE mice. Specifically, GILZ-P inhibits NF-κB, Th-1 cytokines, and T-Bet transcription but increases expression of GATA-3 and Th-2 cytokines, mimicking GILZ (48), and GC activity (17, 27).

GILZ negatively modulates Th-17 development by binding to IL-21 and Irf4 sites, as demonstrated via ChIP-seq analysis of Th-17 cells. These sites overlap the binding sites of major transcription factors involved in Th-17 polarization. Therefore, GILZ may act as a transcriptional repressor, inducing displacement of Th-17 transcription factors from their sites with inhibitory effects on Th-17 development (49). Consistently, GILZ downregulation in naïve CD4+ T cells is required for development of Th-17 (29). GILZ expression in T cells is protective against several pathologies, including psoriasis, a disease commonly treated with GCs, and myocardial infarction, in which Th-17 lymphokines are pathogenetic (29, 50). However, conflicting data were obtained *in vivo* with imiquimod (IMQ), a murine model for IL23-, and IL17-dependent psoriasis. Some researchers demonstrate that IMQ-induced psoriasis is more serious in GILZ-deficient mice, with upregulation of Th-17 cytokines and Th-17 proliferation (29). In contrast, other researchers show that IMQ-induced psoriasis is more severe in GILZ-TG mice, with increased Th-17 cytokines (51) (Figure 2). Thus, based on this model, GILZ can be proinflammatory, similar to the effects of prolonged GC treatment (51), or anti-inflammatory (29).

As mentioned, GILZ can modulate T-cell activity indirectly through its actions on APCs. Its effects on myeloid cells have a broad spectrum of action on all cells of the immune system (9). DC subsets constitutively express GILZ at different levels depending on DC functional status (52). Endogenous GCs appear to regulate constitutive DC GILZ expression, whereas exogenous GCs upregulate DC GILZ *in vivo* and *in vitro*. Thus, by mediating the effects of GCs, GILZ can regulate the balance between activating and tolerogenic DCs (53, 54). GILZ expression is transcriptionally regulated by the GR, which can either induce or inhibit GILZ by recruiting its corepressor, DC-specific transcript (DC-SCRIPT) (Figure 2). Importantly, neutralizing DC-SCRIPT augments GR-induced GILZ expression (55). This suggests that the tolerogenic-promoting effects of GILZ in DCs are so crucial that a biological brake on its expression is required. Indeed, GILZ overexpression induces a DC tolerogenic phenotype comparable to that induced by GCs (56), downregulating the costimulatory molecules CD86, CD83, and CD80 (57, 58), and reducing CD4+ T-cell proliferation (53) (Figure 2). Knocking down GILZ in activated monocyte-derived DCs (Mo-DCs) promotes more efficient CD8+ T-cell secondary responses (59). *In vitro* GC treatment of human Mo-DCs induces GILZ expression, driving a DC tolerogenic phenotype that prevents efficient antigen presentation (57), and induces IL-10-promoting Tregs. Together, these changes inhibit the T-cell response (58). This effect is reproduced by GILZ overexpression (60) and abolished by GILZ silencing (57, 59). Finally, GILZ expression in tumor-infiltrating DCs drives a tolerogenic DC phenotype, and T-cell tolerance against the tumor (54). This suggests that tumor cells may “learn” to secrete GCs to induce GILZ as an escape mechanism against the immune system. These results may explain how GCs, both endogenous and exogenously administered, can either block or worsen tumor progression (especially epithelial tumors) through GILZ expression (61).

Similar to DCs, human monocytes and mouse macrophages constitutively express GILZ. GCs further upregulate GILZ expression, which, via inhibition of NF-κB, mediates GC activity in these cells (62–64) (Figure 2). In fact, transfecting GILZ into THP-1 macrophages mimics the effects of GCs and inhibits the production of chemokines, and costimulatory molecules (62). Correspondingly, GILZ is downregulated by Toll-Like agonists, leading to macrophage activation (65). Moreover, GILZ expression is decreased during neuroinflammation, inversely correlating with the development of innate immune responses (66), and in white blood cells from patients with sepsis (67). These findings confirm the immunosuppressive role of GILZ in myeloid cells and the biological necessity of GILZ downregulation for efficient natural or adaptive immune responses.

Furthermore, expression of GILZ, as with GC (15), limits Th-17 differentiation, and induced Treg cell activity by modulating cytokine production by DCs and mesenchymal cells (68, 69). In a mouse model of rheumatoid arthritis, GILZ expression in mesenchymal stem cells (MSCs) is required for therapeutic effectiveness of MSCs in arthritis (68) and inhibition of transferred- Th-1, and Th-17 cells in immunized mice (70). In a model of acute kidney injury, TAT-GST-GILZ fusion protein conferred renoprotection by regulating cross-talk between T cells and neutrophils, reducing proinflammatory type 1 neutrophils and Th-17 cells, and increasing anti-inflammatory type 2 neutrophils and Tregs (71).

The role of GILZ in T-cell activation is even more complex if we consider the effects on its expression following accessory molecule triggering. Indeed, blocking the co-accessory molecule, CD80, enhances GILZ expression in activated CD4+ T cells (72). However, this field of investigation remains unexplored.

## Perspective and Expectations

Based on our critical review of the literature, we suggest that GILZ has at least three different functions in T cells: (1) endogenous; (2) mediator of GC activity; and (3) as a drug.

As discussed above, basal endogenous GILZ expression in immune cells has a predominant role in T-cell activation, the development of CD4+ naïve T cells, and the physiological control of inflammation. The latter is demonstrated by the many murine models of inflammation, in which the absence of GILZ aggravates inflammatory pathologies (12, 29, 32, 42, 47). However, GILZ expression in T cells underlies many of the effects of GCs established in experimental *in vivo* and *in vitro* models. These models demonstrate that a lack of GILZ inhibits the activity of GCs, and overexpression may mimic GC effects (8–10, 43).

The use of GILZ as a drug is a great challenge given the potential side effects on metabolism. However, many experimental models support and encourage this possibility. Experiments with fusion proteins TAT-GST-GILZ, and HHph-GILZ, viral constructs GILZ-rAAV expressing GILZ, and GILZ-peptide GILZ-P provide examples of achieving pharmacokinetic, pharmacodynamic, and therapeutic efficacy using GILZ *in vivo* as a drug (29, 32, 45, 47, 73). Many of the experimental models discussed above involve pathologies due to an imbalance of the development of naïve CD4+ cells, demonstrating how the therapeutic activity of GILZ is related to actions on T cells (11, 12, 45, 47).

GCs inhibit T-cell activation through genomic and non-genomic mechanisms. GR-mediated genomic regulation induces immunosuppressive molecules, including GILZ (8, 15, 74–76). GCs also modulate T-cell activity through non-genomic mechanisms that occur immediately after drug exposure (77, 78). In T cells, the GR physically associates with the TCR in a multiprotein complex with LCK, and FYN. Short-term treatment with DEX induces the non-genomic destruction of this complex, thereby limiting TCR activation (20) (Figure 1). Is it possible to hypothesize that GCs regulate GILZ function and/or expression through both genomic and non-genomic mechanisms? The regulation of GILZ by GC non-genomic effects would lay the groundwork for several future lines of study. In particular, because GC-induced GILZ transcription in T cells interacts with and inhibits TCR-triggered signaling pathways and transcription factors, it is likely that there is a GC-induced non-genomic effect on constitutive GILZ expression. This would reveal another mechanism by which GCs regulate the T-cell response. Such a mechanism might provide further explanation for the basal level of GILZ in immune cells (63, 79). Therefore, it would be interesting to investigate whether GC/GR interactions induce rapid changes in the cytoplasmic basal pool of GILZ, as such GILZ expression may have alternative functions compared to those of peak GILZ activation induced by GR-mediated transcription. Ultimately, building on our understanding of the molecular mechanisms involving GCs and GILZ may improve the use of GCs as clinical therapeutics and limit treatment-related side effects.

## Author Contributions

EA wrote the review and suggested the general topic. SA prepared the figures. LC and DD discussed and reviewed the manuscript. CR suggested the general topic and discussed and reviewed the manuscript.

### Conflict of Interest Statement

The authors declare that the research was conducted in the absence of any commercial or financial relationships that could be construed as a potential conflict of interest.
